# Effect of *Taraxaci Herba* on Bone Loss in an OVX-Induced Model through the Regulation of Osteoclast Differentiation

**DOI:** 10.3390/nu14204354

**Published:** 2022-10-18

**Authors:** Jun Heo, Minsun Kim, Jae-Hyun Kim, Hwajeong Shin, Seo-Eun Lim, Hyuk-Sang Jung, Youngjoo Sohn, Jaseung Ku

**Affiliations:** 1 Department of Anatomy, College of Korean Medicine, Kyung Hee University, 26, Kyunghee dae-ro, Dongdaemun-gu, Seoul 02447, Korea; 2 Bogwang Korean Medical Clinic 236, Gucheonmyeon-ro, Gangdong-gu, Seoul 05334, Korea

**Keywords:** *Taraxaci Herba*, osteoclast, RANKL, NFATc1, c-Fos, ovariectomized

## Abstract

Bone is a dynamic tissue that maintains homeostasis with a balance of osteoclasts for bone resorption and osteoblasts for bone formation. Women are deficient in estrogen after menopause, which promotes bone resorption due to excessive activity of osteoclasts, leading to osteoporosis. TH (also known as dandelion) is native to warm regions and has traditionally been used to treat gynecological diseases and inflammation. Menopause is a major cause of osteoporosis as it causes abnormal activity of osteoclasts, and various studies have shown that anti-inflammatory drugs have the potential to treat osteoporosis. We analyzed the effect of TH on osteoclast differentiation and the relevant mechanisms using RANKL. After administration of TH in a menopause-like rat model in which ovariectomy of the was rats carried out, changes in bone microstructure were analyzed via micro-CT, and the antiosteoporosis effect of TH was verified by a histological analysis. In addition, the pharmacological effects of TH in an animal model of osteoporosis were compared and analyzed with osteoporosis medications (17β-estradiol (E_2_) and alendronate (ALN)). TH significantly inhibited the initial osteoclast differentiation via the NFATc1/c-Fos mechanism. In addition, bone density in the femur of osteoporotic rats was increased, and the expression of osteoclast-related factors in the serum and tissues was controlled. The results of this study provide objective evidence of the inhibitory effect of TH on osteoclastogenesis and OVX-induced bone loss.

## 1. Introduction

As we enter an aging society due to the development of the medicine and economy, the number of osteoporosis patients is rapidly increasing [1,2]. Osteoporosis is known as “a disease of the skeletal system in which bones become brittle due to a decrease in microstructure and bone mass” [3]. The World Health Organization (WHO) criterion is defined as a bone mineral density (BMD) (T-score < −2.5 SD) that is at least 2.5 standard deviations lower than the average of young, healthy women [4]. Bone is a dynamic tissue that maintains homeostasis with a balance of osteoclasts for bone resorption and osteoblasts for bone formation. Women are deficient in estrogen after menopause, which promotes bone resorption due to excessive activity of osteoclasts, leading to osteoporosis [5]. Osteoporosis increases vulnerability to fractures due to decreased BMD. Furthermore, osteoporotic fractures impose an economic burden and are associated with increased mortality [6]. Osteoporosis is classified into primary and secondary types according to the cause. Among these, primary osteoporosis is mainly caused by menopause and aging. Secondary osteoporosis is known to be caused by an underlying disease or medication [7]. According to previous studies, the prevalence of osteoporosis is estimated to be one-third of the population aged 50 to 60, and is expected to increase to more than 50% of the population aged 80 and over [8,9]. Osteoporosis treatment is divided into bone resorption inhibitors and bone formation promoters (anti-resorptives and anabolic stimulators) [10]. Currently, bone resorption inhibitors are chiefly used for the treatment of osteoporosis [11]. However, it has been found that long-term treatment with bone resorption inhibitors causes side effects such as jaw bone necrosis [12], musculoskeletal pain [13], atypical fractures [14], fatigue, hot flashes, and atrophic vaginitis [15]. In addition, previous studies have suggested that treatment with bisphosphonates in patients at high risk for fractures may have adverse effects on fracture treatment due to changes in osteoclast function [16]. Therefore, the development of alternative drugs with fewer side effects while inhibiting osteoclasts is important.

Osteoclasts are derived from mononuclear myeloid precursors [17,18]. The major cytokine mediating differentiation into osteoclasts is receptor activator of nuclear factor-κB (NF-κB) ligand (RANKL). RANKL binds to receptor activator of NF-κB (RANK) expressed on the surface of osteoclast progenitors to induce osteoclast differentiation-inducing factor [19,20]. Thereafter, c-Fos activates another transcription factor, nuclear factor of activated T cells 1 (NFATc1). Subsequently, activated NFATc1 increases the expression of genes involved in osteoclast differentiation, formation, and resorption [21,22]. 

A member of the Asteraceae (Compositae) family, *Taraxaci Herba* (TH), popularly referred to as “dandelion”, grows in warmer temperate zones and is mainly distributed in Asia, Europe, and the USA [23]. In Korean medicine, it has been reported to be used to treat fever, to reduce edema, and to treat gynecological diseases, in particular [24,25]. In North America, TH is mainly used in folk remedies, and it is also used primarily in traditional Chinese medicine for its health benefits [26]. It is also known as a non-toxic herb used in traditional medicine for its anti-oxidant, anti-inflammatory, and diuretic properties [24,27]. TH is also an edible plant, the leaves, roots, and flowers of which are used in a variety of foods. 

Previous studies have shown that inflammation is associated with metabolic bone disease [28]. TH and components in TH have been shown to have various effects, such as anti-inflammatory effects. The leaves of TH have been found to have anti-inflammatory effects [29], and the representative components of TH are known flavonoids such as caffeic acid, chlorogenic acid, luteolin, and luteolin-7-O-glucoside [24,30]. Among these, caffeic acid has been found to improve skin edema by inhibiting the expression of tumor necrosis factor-α (TNF-α), interleukin 6 (IL-6), and interleukin-1β (IL-1β) in in vivo models [31]. In addition, chlorogenic acid has been found to exhibit anti-inflammatory effects in lipopolysaccharide (LPS)-activated RAW 264.7 cells [32]. Luteolin and luteolin-7-O-glucoside have been found to inhibit nitric oxide (NO) and prostaglandin E2 (PGE2) in LPS-activated RAW 264.7 cells [33]. Commonly, inflammation has been found to promote bone resorption, and TNF-α not only promotes RANKL production but also has a particularly potent effect on osteoclastogenesis. A recent study demonstrated that IL-1 mediates the osteoclastogenic effect of TNF-α by enhancing the stromal cell expression of RANKL and by directly stimulating the differentiation of osteoclast precursors. Furthermore, one of the mechanisms by which TNF-α inhibits osteoblast differentiation is inhibiting Smad signaling in osteoblast differentiation through an NF-κB-mediated process [34]. Inhibition of inflammatory cytokines is an important method in the treatment of osteoporosis [28,35]. In addition, according to previous studies, it can be confirmed that caffeic acid [36], chlorogenic acid [37], and luteolin [38] have osteoclast inhibitory effects. Based on previous research, we speculated that TH could be effective in inhibiting osteoclast differentiation. The effects of TH on osteoclast differentiation in cells and postmenopausal osteoporosis in animals have not been studied. Therefore, we tested the hypothesis that TH could decrease osteoclast differentiation and prevent bone loss in an animal model of postmenopausal osteoporosis by suppressing the c-Fos/NFATc1 mechanism. In addition, the pharmacological effects of TH in an animal model of osteoporosis were compared and analyzed with osteoporosis medications (17β-estradiol (E_2_) and alendronate (ALN)).

## 2. Materials and Methods

### 2.1. Reagents

RAW 264.7 cells were purchased from the Korean Cell Line Bank (Seoul, Korea). Fetal bovine serum (FBS) was obtained from Atlas Biologicals (Fort Collins, CO, USA). Penicillin/streptomycin (P/S), minimum essential medium eagle alpha-modification (α-MEM), and normal serum were purchased from Gibco (Gaithersburg, MD, USA). E_2_ and ALN were supplied by Sigma-Aldrich (St. Louis, MI, USA). Osteo assay surface multiple-well plates were supplied by Corning Inc. (Corning, NY, USA). Nitrocellulose membranes were purchased from Bio-Rad Laboratories, Inc. (Hercules, CA, USA). Taq polymerase was purchased from Kapa Biosystems (Woburn, MA, USA). Primary antibodies against anti-β-actin (cat no: 8432) and anti-c-Fos (cat no: sc-447) were supplied by Santa Cruz Biotechnology, Inc. (Santa Cruz, CA, USA). Anti-NFATc1 (cat no: 556602) was supplied by BD Pharmingen, Inc. (San Diego, CA, USA), and secondary antibodies were purchased from Jackson ImmunoResearch Laboratories, Inc. (West Grove, PA, USA).

### 2.2. Preparation of TH

TH was supplied by Omni Herb Inc. (Seoul, Korea). TH (dried sample: 150 g) was extracted in 80% Et-OH at 4 °C for 3 weeks, and the extract was concentrated using a vacuum concentrator (EYELA, Tokyo, Japan). Thereafter, the dried powder (yield ratio: 9.5%) was obtained using a freeze-dryer and kept at −20 °C for use in the experiment.

### 2.3. Liquid Chromatography–Mass Spectrometry (LC–MS) Analysis for Marker Components

LC–MS analysis of maker compounds in TH extract was conducted with a Waters e2695 system (ACQUITY QDa. Waters Corporation, Milford, MA) and Sunfire C18 (250 × 4.6 mm, 5 μm) column. Acetonitrile (A) and water with 0.1% formic acid (B) were used as mobile phases; the flow rate was 0.3 mL/min and the injection volume was 10 μL. Gradient program conditions were 0 min, 5% B; 0–30 min, 5–50% B; 30–60 min, 50–95% B. MS conditions were as follows: ESI mode, the capillary voltage was set to 0.8 kV, and the cone voltage was 15 V (+) or 30 V (−). MS data were acquired in the positive scan mode (mass range m/z 100–600 Da).

### 2.4. Cell Culture and Cytotoxicity

RAW 264.7 cells were cultured as previously described [25]. The cell counting kit-8 (CCK-8, Dojindo, Tokyo, Japan) was used according to the manufacturer’s instructions to examine the cytotoxicity of TH. Briefly, RAW 264.7 cells were seeded and incubated overnight. Next, the cell treated with Dulbecco’s modified eagle medium (DMEM, without FBS) and various concentrations of TH (25, 50, 100, 200 μg/mL). The cells were incubated with 20 μL of CCK-8 solution at 37 °C for 2 h. 

To assess the osteoclast cytotoxicity of TH, RAW 264.7 cells were seeded, and the cells were treated with a RANKL (PeproTech, London, U.K.; 100 ng/mL) and TH (25, 50, 100, and 200 μg/mL) for 5 days. After, the cells were incubated with 20 μL of CCK-8 solution at 37 °C for 2 h. Cell cytotoxicity was analyzed by an enzyme-linked immunosorbent assay (ELISA) reader (VersaMax microplate reader, Molecular Devices, LLC).

### 2.5. Tartrate-Resistant Acid Phosphatase (TRAP) Staining and TRAP Activity

The differentiated osteoclasts were stained using the TRAP staining kit (Sigma-Aldrich, St. Louis, MI, USA). Briefly, RAW 264.7 cells were treated with a differentiation medium for 5 days for differentiation of osteoclasts (differentiation medium: α-MEM including RANKL (100 ng/mL) and TH (25, 50, 100, and 200 μg/mL). 

To determine the effect of the time-point treatment of TH on osteoclast differentiation, cells were inoculated as previously described and treated with TH by period. Then, the cells were fixed with 10% formalin and stained using the TRAP staining kit. TRAP-positive cells were counted using a microscope (only cells with three or more nuclei were counted, magnification ×100). To analyze the effect of TH on TRAP activity, 50 μL of differentiation medium was transferred to a new plate. The TRAP solution was prepared and dispensed according to a previous study using the same volume of the TRAP solution and incubated. TRAP activity was stopped and analyzed by an ELISA reader.

### 2.6. F-Actin Ring Formation and Pit Formation Assay

The F-actin ring was stained using the 4′,6-diamidino-2-phenylindole (DAPI) and Acti-stain™ 488 Fluorescent Phalloidin. Briefly, cell differentiation was performed as previously described, and differentiation cells were fixed for 20 min. To increase cell permeability, the cells were treated with 0.1% Triton X-100 and Acti-stain™ 488 Fluorescent Phalloidin. Additionally, the cells were stained with DAPI and then counted. DAPI was treated and photographed using a fluorescence microscope (Cellena; magnification, ×200), and the stained cells were counted.

To investigate the effect of TH on pit formation, the cells were inoculated into osteo assay surface multiple-well plates. After 24 h, the cells were treated with a differentiation medium for 5 days and eliminated with 4% sodium hypochlorite.

### 2.7. Western Blot

Total protein was lysed in an radioimmunoprecipitation assay buffer (RIPA buffer, composition of the buffer used: 50 mM Tris-Cl, 150 mM NaCl, 1% NP-40, 0.5% sodium deoxycholate, and 0.1% SDS). Briefly, proteins were collected and quantified. An equal volume of protein samples was loaded into 10% sodium dodecyl sulfate (SDS)-polyacrylamide gel and transferred onto a nitrocellulose membrane for 1 h. Then, 5% skim milk was used to eliminate nonspecific binding for 1 h. The membrane was washed 3 times with Tris-based immunoblot wash buffer (TBST) and incubated with a primary antibody in 1% bovine serum albumin (BSA) in TBST at 4 °C overnight. The primary antibodies used in this study were as follows: NFATc1 and β-actin were diluted 1:1000, and c-Fos was diluted 1:200. After washing the next membrane 3 times, it was incubated with a secondary antibody. An enhanced chemiluminescence (ECL) solution was used to visualize the membrane, and bands were normalized as loading controls.

### 2.8. Reverse Transcription Polymerase Chain Reaction (RT-PCR)

Total RNA was extracted according to the manufacturer’s instructions for TRIzol^®^ reagent (Invitrogen, Carlsbad, CA, USA). Briefly, RAW 264.7 cells were seeded, and the cells were treated with a differentiation medium for 4 days. All the primers for RT-PCR in Table 1 were purchased from Genotech (Daejeon, Korea). RNA was quantified using the NanoDrop 2000 instrument (Thermo Scientific, Cambridge, U.K.), and after synthesizing RNA into cDNA, PCR was performed on the cDNA sample using a C1a000 Touch™ Thermal Cycler. Bands were captured using a NaBI gel document (Neoscience, Suwon, Korea). All bands were measured using ImageJ software Ver. 1.46. Expression levels of the target genes were normalized using the expression level of a housekeeping gene such as β-actin. 

### 2.9. Experimental Protocols

Female Sprague–Dawley (SD) rats (11 weeks old were used in in vivo experiments. The Kyung Hee Medical Center Institutional Animal Care and Use Committee approved the in vivo experimental protocols (approval no. KHMC-IACUC 19-017). The animals were stabilized for 1 week. To establish the ovariectomy (OVX) induction model, the operation was performed under respiratory anesthesia (conditions: 100% oxygen and 5% isoflurane). Then, all the animals had both sides of the dorsal hair removed, and the skin and muscles were incised to expose the ovaries. In the OVX group, both exposed ovaries were excised and the muscle and skin were sutured. The sham group had the same stress applied without excision of the exposed ovaries, and then the muscles and skin were sutured. After surgery, gentamicin (4 mg/kg) was delivered via intraperitoneal injection (IP) for 3 days to prevent infection. 

The experimental groups (*n* = 8) in this study were classified as follows: (1) sham group (oral administration of distilled water (D.W.) after sham operation), (2) OVX group (oral administration of D.W. after OVX-induced operation), (3) E_2_ group (oral administration of 100 μg/kg E_2_ after OVX-induced operation), (4) ALN group (oral administration of 5 mg/kg ALN after OVX-induced operation), (5) TH-L group (oral administration of 12.64 mg/kg after OVX-induced operation), and (6) TH-H group (oral administration of 78.36 mg/kg after OVX-induced operation). The calculation of the dose for the TH group proceeded as follows. According to Korean medicine, the recommended amount for an adult weighing 60 kg is 8 g. Therefore, the dose of TH per 1 kg was 12.64 mg, and 12.64 mg/kg was administered to the TH-L group. In addition, it is known that the metabolism of rats is 6.2 times faster than that of humans [39,40]. On this basis, the TH-H group was dosed with 78.36 mg/kg. During the 8 weeks of the experiment, E_2_, ALN, and TH were liquefied in D.W. and administered. The animals were weighed once a week at fixed times.

In this study, humane endpoints were established to prevent animal pain and distress in experimental animals during surgery and drug administration: (I) if the animals had difficulty consuming food or water due to inconvenient walking, (II) if the body weight was reduced by 20% or more as compared to the normal group, and (III) if the animals fell into an unconscious state and did not respond to external stimuli. No animals exhibited behaviors that corresponded to the humane endpoints during the research period. After the end of the experiment, the animals were anesthetized under the same conditions as before in order to sacrifice all the animals. The conditions for animal sacrifice were as follows. After a lethal dose (10 mL) of cardiac blood was removed on all animals, it was confirmed that the heart had stopped, and the animal was sacrificed by cervical dislocation. After that, uterus and femur samples were collected, and the uterus samples were weighed.

### 2.10. Serum Analysis

The serum levels of aspartate aminotransferase (AST), alanine transferase (ALT), and alkaline phosphatase (ALP) were analyzed by DKKOREA (Seoul, Korea). The serum levels of TRAP were analyzed according to the manufacturer’s protocol. 

### 2.11. Micro-Computed Tomography (Micro-CT) Analysis

Femur samples collected after sacrifice were fastened in 10% neutral buffered formalin (NBF) and rinsed with running water for 24 h. The femoral head was scanned by micro-CT referring to the protocol of a previous study [41] (SkyScan1176; Bruker Corporation; Kontich, Antwerpen, Belgium). Bone microstructural parameters such as BMD, bone volume/total volume (BV/TV), and trabecular separation (Tb.Sp) were also analyzed using NRecon software (Bruker Corporation).

### 2.12. Histological Examination and Immunohistochemistry

Before embedding, femur samples were demineralized using 10% ethylenediaminetetraacetic acid (EDTA) to remove calcium from the bones. After the demineralized samples were dehydrated, they were embedded in paraffin and sectioned to a thickness of 4 μm. Sectioned tissues were dried in a tissue dryer for 1 day. All the sectioned tissue was deparaffinized with xylene and dehydrated with 100%, 95%, 90%, 80%, and 70% Et-OH and washed with D.W. In order to proceed with hematoxylin and eosin (H&E) staining, slides were dehydrated using 70%, 80%, 90%, 95%, and 100% Et-OH and then cleared using xylene and sealed using a mounting solution. The tissues were observed, and images were captured (magnifications: ×40 and ×100). 

To proceed with immunohistochemistry (IHC) staining, the tissues were blocked with endogenous peroxidases using 0.3% hydrogen peroxide (H_2_O_2_). Then, proteinase K (0.4 mg/mL) was reacted at 37 °C for 30 min for antigen retrieval in tissues and 10% normal serum in TBS was used to block unnecessary reactions. Subsequently, the tissues were incubated with the antibody overnight. On the second day, the reaction was performed with an avidin–biotin complex (ABC) kit. Finally, after staining with 3,3-diaminobenzidine (DAB) and hematoxylin, images were captured and observed under a microscope.

### 2.13. Data Statistics and Analysis

All the data are expressed as the mean ± standard error of the mean (SEM) values of three experiments. One-way ANOVA and Tukey’s post hoc test were used to examine the significance of differences between the results; two-way ANOVA was used to verify the change in body weight for treatment × time. *p* < 0.05 was considered significant. The results were statistically analyzed using Graph Prism software (version 5.01, GraphPad Software, Inc., version 5.01, San Diego, CA, USA).

## 3. Results

### 3.1. LC-MS Analysis for TH Extract

**Figure 1:**
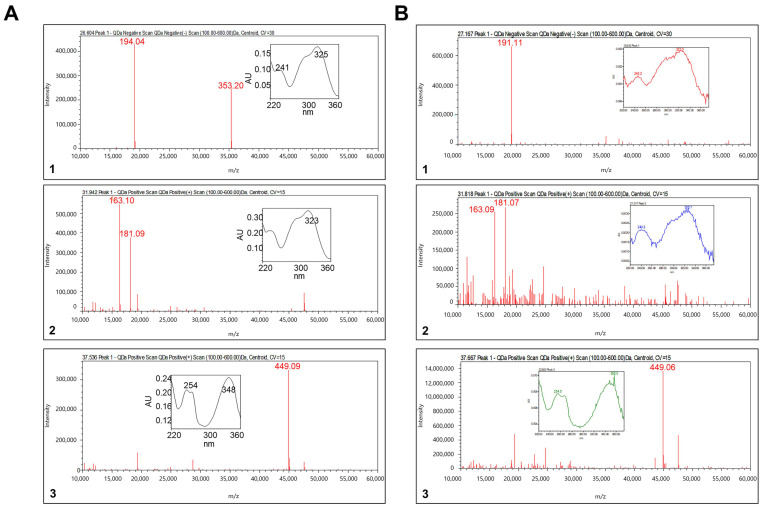
Representative LC-MS result: (**A**) standard solution and (**B**) TH sample. (**1**) Chlorogenic acid, (**2**) caffeic acid, and (**3**) luteolin 7-O-glucoside.

Analysis of the three main components of TH was performed by LC-MS using ACQUITY TQD LC-MS/MS. As shown in Figure 1A, three compounds were detected in the TH extract: (1) chlorogenic acid, (2) caffeic acid, and (3) luteolin 7-O-glucoside.

### 3.2. TH Restrained RANKL-Induced Osteoclast Differentiation in RAW 264.7 Cells

**Figure 2:**
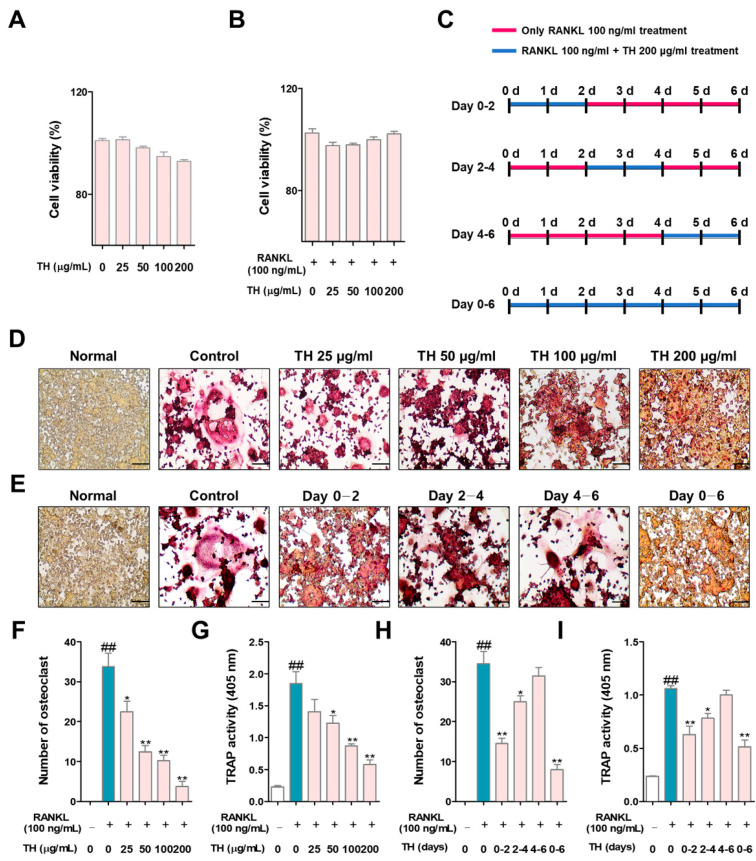
TH treatment decreased nuclear factor-κB (NF-κB) ligand (RANKL)-induced osteoclast differentiation in RAW 264.7 cells. (**A**) To determine the cell cytotoxicity of *Taraxaci Herba* (TH), a cell counting kit-8 (CCK-8) assay was performed. (**B**) The cytotoxicity of TH in osteoclasts was measured using the CCK-8 assay. (**C**) The TH processing schedule for each period was as follows: (**D**) tartrate-resistant acid phosphatase (TRAP)-positive cells were stained with a TRAP staining kit (×100; scale bar, 200 μm); (**E**) TH was used for different durations over six days at four different time phases; (**F**,**H**) TRAP-positive cells were counted using ImageJ software Ver. 1.46; (**G**,**I**) TRAP activity was confirmed at an absorbance of 405 nm. The experiments’ representative images are shown, and the graphs are expressed as the mean ± standard error of the mean (SEM; *n* = 3). ^##^ *p* < 0.01 vs. normal group; * *p* < 0.05, ** *p* < 0.01 vs. control group.

Cumulative studies have reported that TRAP is an important marker in osteoclast differentiation; therefore, we analyzed the effect of TH using TRAP staining. First, we confirmed the cytotoxicity of TH before confirming the osteoclast inhibitory ability. As shown in Figure 2A, TH treatment showed no cytotoxicity at the experimental concentrations of 25, 50, 100, and 200 μg/mL. Furthermore, we confirmed that TH (25, 50, 100, and 200 μg/mL) treatment was not toxic in osteoclasts (Figure 2B). Briefly, these results confirm that TH had an osteoclast inhibitory effect that was not mediated by cytotoxicity. The schedule for the time-course experiment is shown in Figure 2C. To clarify the osteoclast differentiation effect of TH, we further examined the TRAP staining and activity in the RANKL-induced model. We found that the number of TRAP-positive cells and TRAP activity was increased in the RANKL-induced cells, and treatment with TH suppressed the number and activity of osteoclast-positive cells (Figure 2D,F,G). Then, a time-course experiment was performed to confirm the stage in which the osteoclast inhibitory effect of TH is mainly exhibited. As a result of the experiment, TH showed its osteoclast inhibitory effect at various stages, and the stage showing the best effect was at 0–2 days (Figure 2E,H,I). 

### 3.3. TH Suppresses Osteoclast Function and Bone Resorption

**Figure 3:**
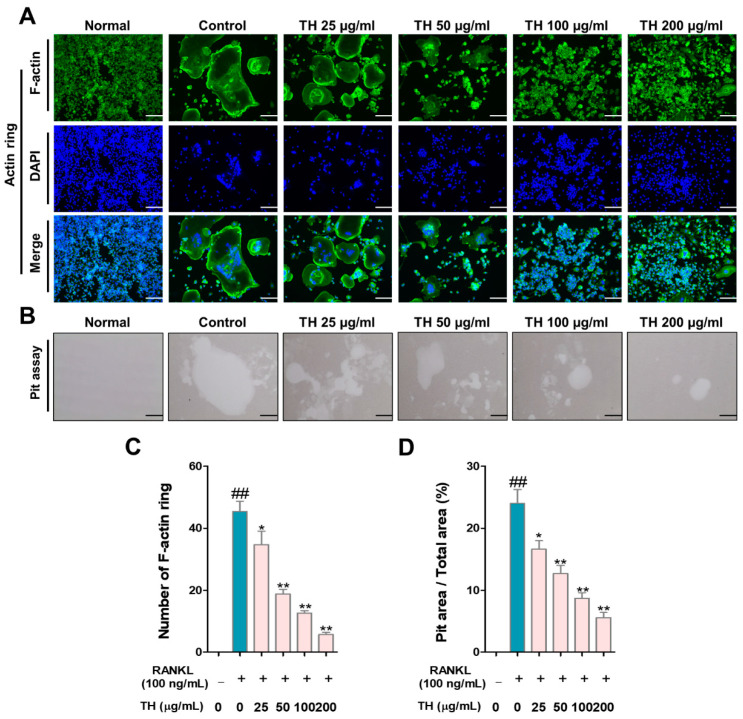
*Taraxaci Herba* (TH) treatment reduced F-actin ring formation, size, and pit area. (**A**) Representative images of F-actin ring (×100; scale bar, 200 μm). (**B**) Representative images of pit area of nuclear factor-κB (NF-κB) ligand (RANKL)-induced osteoclast differentiation in RAW 264.7 cells (×100; scale bar, 200 μm). (**C**,**D**) Measurement of F-actin ring-positive cells and pit area using ImageJ software Ver. 1.46. The experiments’ representative images are shown, and the graphs are expressed as the mean ± standard error of the mean (SEM; *n* = 3). ^##^ *p* < 0.01 vs. normal group; * *p* < 0.05, ** *p* < 0.01 vs. control group.

To further examine the effect of TH on the cytoskeletal structure and bone resorption, we tested the F-actin ring formation and pit formation. As shown in Figure 3A,C, the number and size of F-actin rings were induced in RANKL-induced cells and treatment with TH inhibited the number and size of osteoclast-positive cells. The pit area was increased in RANKL-induced cells and the pit area was reduced after TH treatment (Figure 3B,D).

### 3.4. TH Lower the Expression of Osteoclast Differentiation Transcription Factor

**Figure 4:**
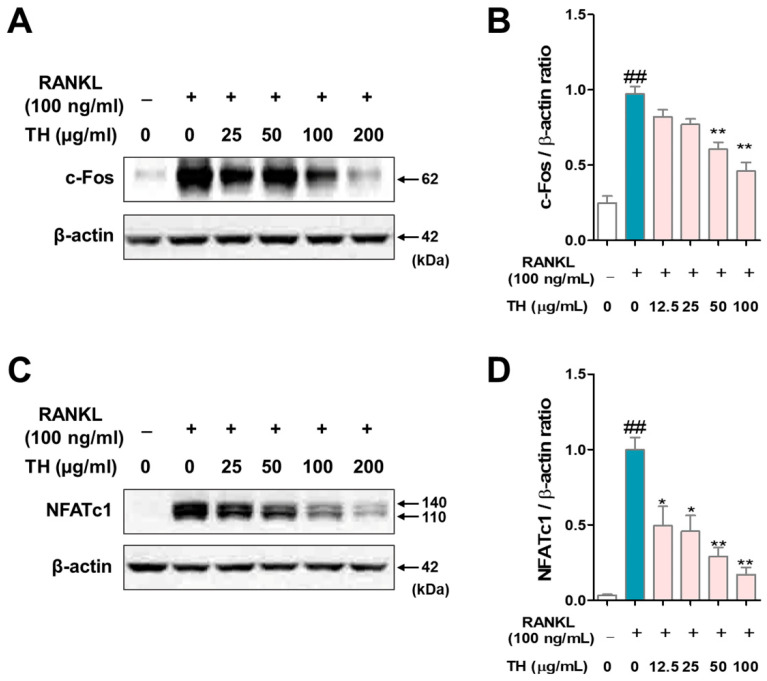
*Taraxaci Herba* (TH) treatment reduced the protein expression of c-Fos and nuclear factor of activated T cells 1 (NFATc1). (**A**) c-Fos expression was analyzed by Western blot. (**B**) c-Fos protein expression was quantified using ImageJ software Ver. 1.46 with *Actb* (β-actin). (**C**) NFATc1 expression was analyzed by Western blot. (**D**) NFATc1 protein expression was quantified using ImageJ software Ver. 1.46 with *Actb* (β-actin). The experiments’ representative images are shown, and the graphs are expressed as the mean ± standard error of the mean (SEM; *n* = 3). ^##^ *p* < 0.01 vs. normal group; * *p* < 0.05, ** *p* < 0.01 vs. control group.

c-Fos and NFATc1 are the key transcription factors in osteoclast differentiation. We used RANKL to stimulate RAW 264.7 cells and evaluated the protein expression of c-Fos and NFATc1 by Western blot. The results showed that the protein expression of c-Fos and NFATc1 were increased in RANKL-induced cells, and treatment with TH suppressed the protein expression of c-Fos and NFATc1 (Figure 4A–D). 

### 3.5. TH Lowers the Expression of Osteoclast-Related Genes by Inhibiting the Expression of Transcription Factors Such as c-Fos and NFATc1

**Figure 5:**
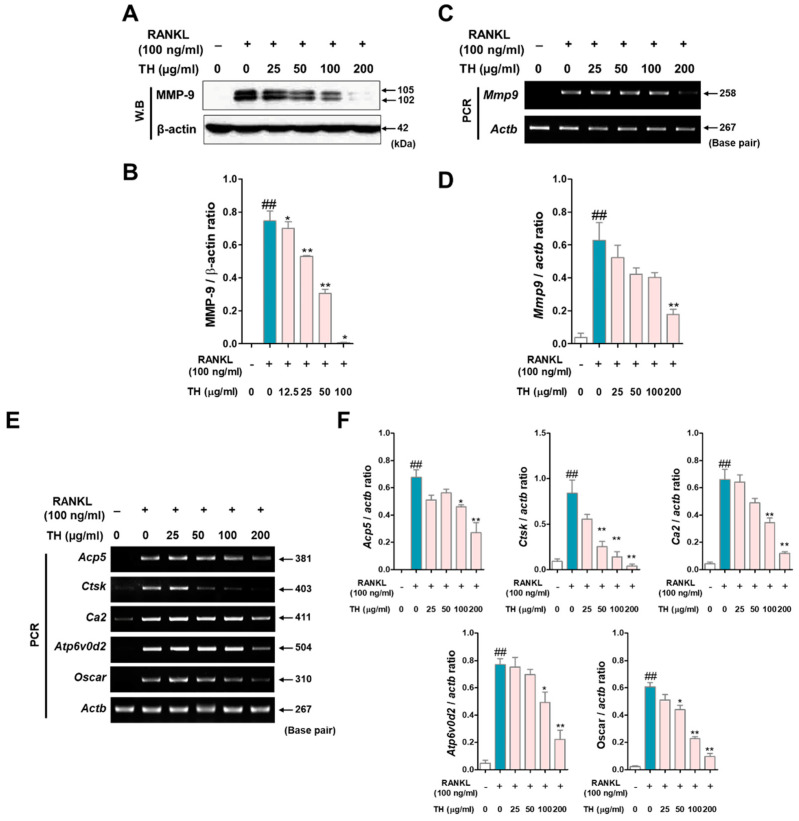
*Taraxaci Herba* (TH) treatment reduced the mRNA expression of osteoclast-related genes. (**A**) MMP-9 expression was analyzed by Western blot. (**B**) Protein expression was quantified using ImageJ software Ver. 1.46 with *Actb* (β-actin). (**C**) Matrix metallopeptidase 9 (*mmp9,* MMP-9) expression was analyzed by real time reverse transcription polymerase chain reaction (RT-PCR). (**D**) mRNA expression was quantified using ImageJ software Ver. 1.46 with *Actb* (β-actin). (**E**) mRNA expressions of tartrate-resistant acid phosphatase (TRAP) (*Acp5,* TRAP), cathepsin K (*Ctks*, CTK), (*Ca2,* CA2), atpase H+ transporting V0 subunit d2 (*Atp6v0d2,* ATP6v0d2), and osteoclast-associated receptor (*Oscar*, OSCAR) were measured using RT-PCR. (**F**) *Acp5*, *Mmp9*, *Ctks*, *Ca2*, *Atp6v0d2,* and Oscar mRNA expressions were quantified using ImageJ software Ver. 1.46. with glyceraldehyde 3-phosphate dehydrogenase (*Gapdh,* GAPDH). The experiments’ representative images are shown, and the graphs are expressed as the mean ± standard error of the mean (SEM; *n* = 3). ^##^ *p* < 0.01 vs. normal group; * *p* < 0.05, ** *p* < 0.01 vs. control group.

Next, to evaluate whether TH has an effect on the mRNA expression of osteoclast-related genes, we examined this using Western blot and RT-PCR. As a result of analyzing the expression of matrix metalloproteinase-9 (MMP-9) by Western blot and RT-PCR, the protein and mRNA expression of MMP-9 were upregulated in RANKL-induced cells, and treatment with TH decreased the protein and mRNA expression (Figure 5A–D). Moreover, we found that the expression of osteoclast-related genes was increased in RANKL-induced cells, and treatment with TH suppressed the expression (Figure 5E,F). 

### 3.6. Changes in Body Weight and Effects of TH on Serum Levels

**Figure 6:**
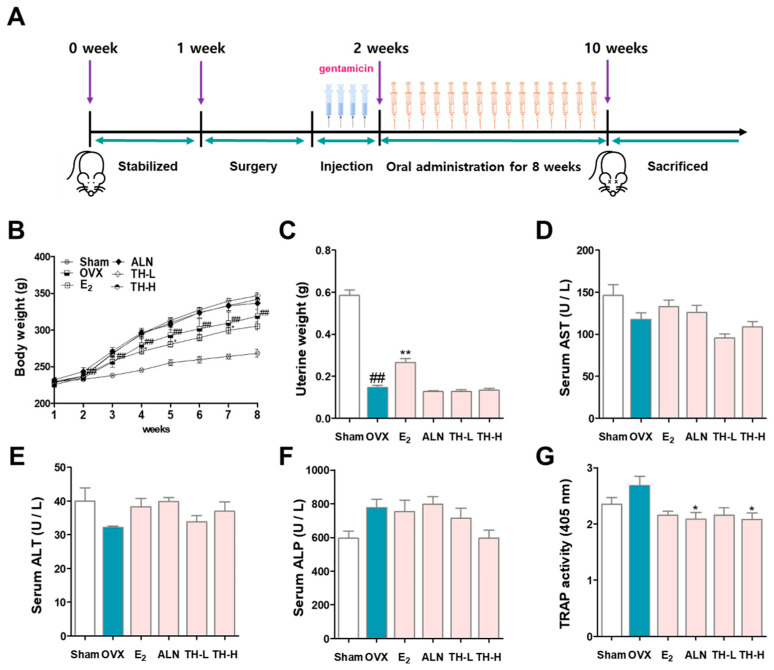
*Taraxaci Herba* (TH) administration improved bone reduction in the ovariectomy (OVX)-model. (**A**) Schedule of in vivo experiments for OVX-induced osteoclast differentiation in the SD rats. (**B**) All the animals were weighed once weekly at the same time. (**C**) At the time of sacrifice, uterine weights were taken and weighted. The levels of (**D**) aspartate aminotransferase (AST), (**E**) alanine aminotransferse (ALT), (**F**) alkaline phosphatase (ALP) and (**G**) tartrate-resistant acid phosphatase (TRAP) in the serum were analyzed through enzyme-linked immunosorbent assay (ELISA). The experiments’ representative images are shown, and the graphs are expressed as the mean standard error of the mean (SEM; *n* = 8). ^##^ *p* < 0.01 vs. sham group; * *p* < 0.05, ** *p* < 0.01 vs. OVX group.

Figure 6A shows the schedule of the in vivo experiment performed in this study. The effect of TH on changes in body weight and uterine weight was investigated. The weight change in each experimental group is shown in Figure 6B. Compared with the sham group, the OVX rats gained weight significantly in a time-dependent manner from 2 weeks after the procedure, and the E_2_ treatment group had significantly inhibited weight gain compared to the OVX group at 4, 5, and 7 weeks. In addition, the ALN, TH-L, and TH-H groups continued to gain weight, but the difference was not significant compared to the OVX group. Furthermore, the uterus weight of the OVX group was significantly lower than that of the sham group. On the other hand, E_2_ administration significantly increased uterine weight, but ALN and TH-L and TH-H administration did not affect uterine weight (Figure 6C). Subsequently, to examine the effect of TH, a serum analysis was performed. The results showed that the OVX, E_2_, ALN, TH-L, and TH-H groups did not demonstrate any AST or ALT changes (Figure 6D,E). As a result of the ALP analysis, the ALP of the OVX group was greater than that of the sham group, and the ALP of the TH-L and TH-H groups was lower than that of the OVX group. On the other hand, the E_2_ and ALN groups showed no effect regarding ALP changes (Figure 6F). Serum TRAP levels of the OVX group were greater than those of the sham group, and the levels in the E_2_, ALN, and TH-L and TH-H groups were lower than those of the OVX group (Figure 6G). 

### 3.7. TH Improved Bone Loss in the Postmenopausal Osteoporosis Rat Model

**Figure 7:**
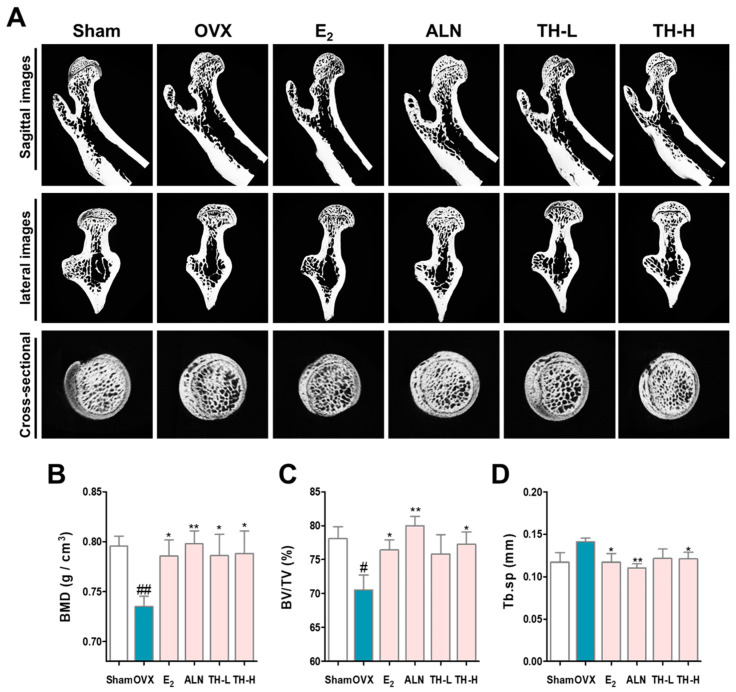
*Taraxaci Herba* (TH) increased the decreased bone density. (**A**) Representative micro–computed tomography (micro-CT) images of the femurs. Relative bone microstructure changes in (**B**) bone mineral density (BMD), (**C**) bone volume/total volume (BV/TV), and (**D**) trabecular separation (Tb.Sp) were measured using NRecon software. The experiments’ representative images are shown, and the graphs are expressed as the mean ± standard error of the mean (SEM; *n* = 8). ^#^ *p* < 0.05, ^##^ *p* < 0.01 vs. sham group; * *p* < 0.05, ** *p* < 0.01 vs. OVX group.

To determine whether TH prevented bone loss effects, we analyzed the femurs using micro-CT (Figure 7A). BMD and BV/TV were significantly decreased by OVX, as shown in the sagittal, lateral, and cross-sectional images, whereas administration with E_2_, ALN TH-L, and TH-H groups resulted in greater BMD and BV/TV than the OVX group (Figure 7 B,C). Tb.sp was greater in the OVX group, and E_2_, ALN, TH-L, and TH-H treatment prevented bone loss (Figure 7D).

### 3.8. TH Increased the Trabecular Area and Reduced the Number of NFATc1 and c-Fos in Femur Tissue

**Figure 8:**
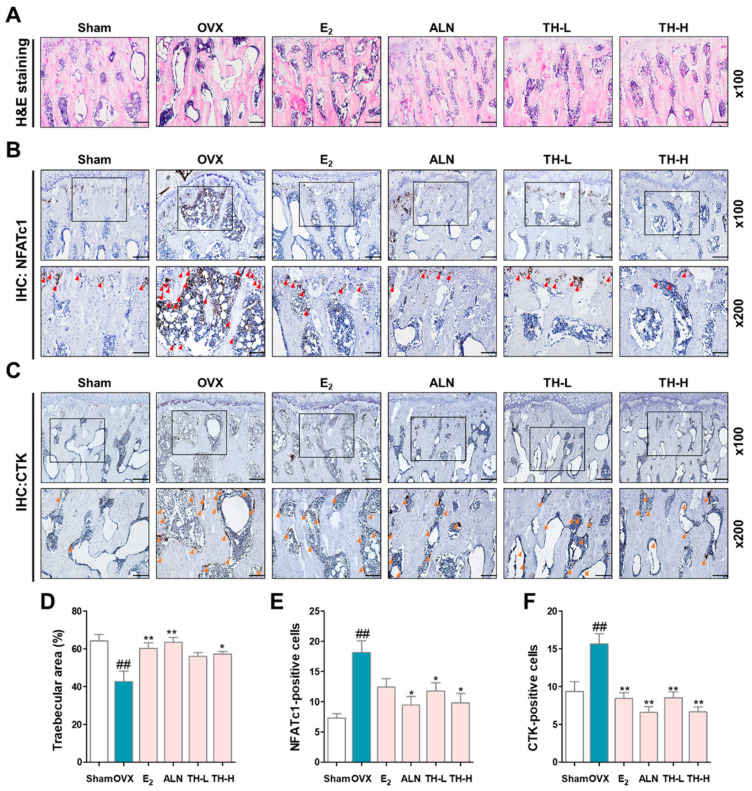
*Taraxaci Herba* (TH) treatment attenuated the trabecular area and expression of nuclear factor of activated T cells 1 (NFATc1) and cathepsin K (CTK) in bone tissue. (**A**) Representative hematoxylin and eosin (H&E) images of femur tissue. (**B**,**C**) Representative immunohistochemistry (IHC) staining images used to assess NFATc1 (red arrows) and CTK (orange arrows). (**D**) The trabecular area was measured using ImageJ software. (**E**,**F**) Quantification of NFATc1- and CTK-positive cells counted using ImageJ software Ver. 1.46. The experiments’ representative images are shown, and the graphs are expressed as the mean ± standard error of the mean (SEM; *n* = 8). ^##^ *p* < 0.01 vs. sham group; * *p* < 0.05, ** *p* < 0.01 vs. OVX group.

To investigate the effect of TH on the reduction in the trabecular area, we performed histological staining using a hematoxylin–eosin staining solution. The trabecular area of the OVX group was lower than that of the sham group, and the trabecular areas in the E_2_, ALN, and TH groups were greater than in the OVX group (Figure 8A,D). Subsequently, to examine whether TH has an effect on the expression of NFATc1 and CTK in femur tissue, we performed IHC staining (Figure 8B,C). As shown in Figure 8E,F, the numbers of NFATc1 and CTK were significantly increased due to OVX, and the numbers of NFATc1 and CTK in the E_2_, ALN, and TH-L and TH-H groups were lower than the OVX group. In addition, the ALN and TH-H groups were significant (Figure 8E,F).

## 4. Discussion

In this research, we have illustrated the effect of TH on osteoclast differentiation and function in vitro by inhibiting RANKL-induced osteoclast differentiation. Additionally, we have revealed the effect of TH on bone loss in an OVX-induced model in vivo. Our trial presented a total of four new findings, and, according to these results, TH has the possibility of being used as a new alternative treatment for osteoporosis. First, osteoclast differentiation, formation, fusion, and bone resorption by RANKL after TH treatment were significantly reduced. Second, the protein expressions of the transcription factors were considerably decreased after TH treatment. In addition, the mRNA expression of osteoclast-related genes was reduced after TH treatment. Third, TH suppressed OVX-induced bone loss after TH treatment. Fourth, TH inhibited the decreased trabecular area in the femur in the OVX model and significantly reduced NFATc1- and CTK-positive cells.

Osteoclasts are multinucleated cells formed by the cytoplasmic fusion of mononuclear precursors and play a role in removing the bone trabeculae formed below the growth plate [42,43]. RAW 264.7 cells are known to be a suitable model of macrophages and are known to readily differentiate into osteoclasts upon exposure to RANKL [44]. Thus, RAW 264.7 cells have been proven to have an important role in osteoclast experiments. RANKL is an essential cytokine inducing osteoclast formation and the maturation of macrophages. TRAP is secreted in large amounts by osteoclasts and is considered an important chemical marker, and its concentration in serum is used as a biochemical indicator of osteoclast function and bone resorption. TRAP activity is enhanced in specifically activated macrophages such as alveolar macrophages [45]. According to the results of our study, TH significantly inhibited osteoclast differentiation. In addition, in the time-course analysis, TRAP-positive mature osteoclasts were found to be fewest in the initial (1–2 days) stage of TH treatment. Osteoclasts make integrin-based contacts with the bone surface, which bind with actin to form a ring-shaped seal area called the F-actin ring. In addition, a representative method used to determine the bone resorption activity of osteoclasts is the pit formation assay. In this study, TH significantly decreased the number of F-actin rings. These data showed that TH decreased the number of TRAP-positive cells, pit formation, and the number of F-actin rings.

RANK binds to RANKL and activates TNF receptor-associated factor 6 (TRAF6), NF-κB, and mitogen-activated protein kinase (MAPK). Then, c-Fos, a transcription factor for osteoclast differentiation, is activated, and c-Fos induces another transcription factor, NFATc1. Previous studies have shown that c-Fos is a key regulator of macrophage and osteoclast lineages, and c-Fos-deficient mice exhibit osteopetrosis. NFATc1 is upregulated during RANKL-induced osteoclastogenesis. In addition to the important role of NFATc1 in osteoclast differentiation, recent studies have shown that NFATc1 is also involved in osteoclast functions such as bone resorption [46]. Additionally, NFATc1-deficient mice develop osteopetrosis due to impaired osteoclast formation, and NFATc1 ectopic expression can induce osteoclast differentiation even in the absence of RANKL [22]. These findings suggest that TH inhibits osteoclast differentiation, formation, and fusion through inhibition of the NFATc1/c-Fos signaling pathway. 

**Figure 9:**
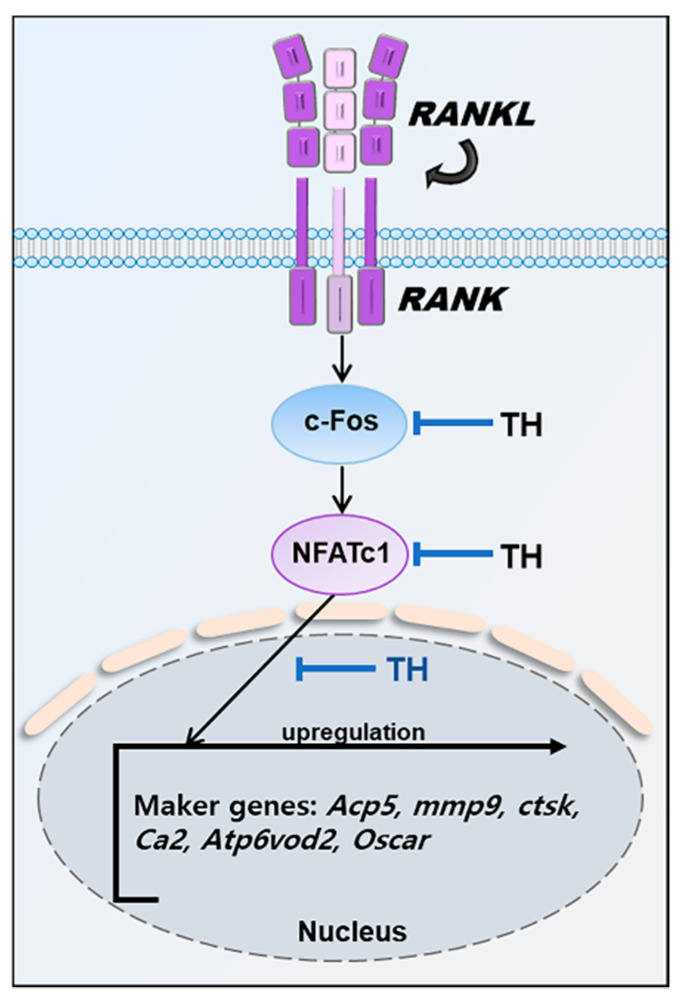
Mechanisms by which TH inhibits osteoclast differentiation.

In particular, NFATc1, a master regulator of osteoclast differentiation, regulates a number of osteoclast-specific genes such as *Mmp9* (MMP-9), *Ctsk* (CTK), *Ca2* (CA2), ATPase H+transporting V0 subunit d2 (Atp6v0d2/ATP6v0d2), and Oscar (OSCAR) in cooperation with c-Fos. *Mmp9* is a major degrading enzyme related to the bone matrix and is involved in bone resorption within the corrugated border; *Mmp9*-deficient mice show delayed fracture recovery [47]. During bone resorption, Ctsk is secreted into the sealing area and the cell matrix adhesive structure of osteoclasts; *Ctsk*-deficient mice display an osteofossil phenotype with excessive trabeculae of the bone marrow space [48,49]. In cell fusion and cell division, intracellular pH plays an important role. The expression of *Ca2* appears in the early stage of osteoclast differentiation, and changes in pH value can be detected by regulating the secretion of H+, which is responsible for bone resorption by osteoclasts. Previous studies have shown that *Ca2* deficiency is associated with osteopetrosis. Cell–cell fusion is essential for the development of multinucleated cells such as macrophages and osteoclasts, and *Atp6v0d2* is a factor involved in cell fusion. Previous studies have shown that *Atp6v0d2*-deficient mice are incapable of impaired osteoclast fusion [50]. Osteoclast-associated receptor (*Oscar*/OSCAR) represents a co-stimulatory signal required for osteoclast differentiation and activation. The interaction between OSCAR and collagen may play an important role in the normal development, maintenance, and repair of bones [51]. In this study, TH suppressed the expression of osteoclast-related genes induced by RANKL. These results suggest that the inhibition of osteoclast differentiation and formation occurs via the NFATc1 signaling pathway (Figure 9).

The postmenopausal osteoporosis model can be used to study bone loss due to estrogen deficiency, and the most commonly used model is the OVX-induced osteoporosis model [52,53]. Rat bone is a tissue that exhibits characteristics similar to those of human bone, and is constructed and reconstructed throughout life in the process of bone remodeling [54]. The OVX osteoporosis model exhibits symptoms of weight gain and bone loss due to estrogen deficiency similar to those of clinical trials. After 2 weeks of the experiment, the body weight of the OVX group was significantly greater than that of the sham group. The body weight of the E_2_ group was lower than that of the sham group. These results suggest that the OVX model is reliable [55]. 

In this study, AST and ALT, liver toxicity-related indicators, were evaluated in serum to determine if TH administration was toxic to the liver. In general, herbal products are natural products and originated thousands of years ago, so there is a common belief that they will be harmless, but there are reports of liver toxicity from time to time. Recently, the public has expressed concern that natural product-based therapeutics may be toxic to the liver, and we wanted to remove these concerns and clearly demonstrate the efficacy and safety of TH [56]. AST and ALT are released into the serum during liver damage and are generally important biochemical markers used in the diagnosis of liver disease. Abnormal liver enzyme levels play a role in indicating liver cell damage [57,58]. According to the results of this study, the OVX, E_2_, ALN, and TH groups showed no effects regarding AST and ALT changes, indicating that E_2_, ALN, and TH dosing had no effect on liver damage. In addition, according to a previous study, TH was found to have hepatoprotective action [59], and similarly, in our study, the TH group had significantly lower AST and ALT levels. ALP is produced in the early stage of differentiation of specific cells called osteoblasts involved in bone formation and is a representative indicator related to bone formation. When bone metabolism is accelerated, osteoblast activity is also increased along with osteoclast activity [60,61]. According to the results of this study, the ALP of the OVX group was greater than that of the sham group and the TH-H group was lower than that of the OVX group. These results indicate that TH inhibited the increased ALP level due to excessive osteoclast activity. In addition, TH suppressed the expression of TRAP, which was upregulated by the abnormal activity of osteoclasts generated by OVX.

Osteoporosis causes a decrease in bone strength, which increases the risk of fractures. According to previous studies, micro-CT has become a standard tool for the measurement and visualization of bone structures [62]. In addition, micro-CT is known to be capable of performing three-dimensional quantification of bone structure and can be used to measure microstructures reflecting BMD and cancellous bone. BV/TV, also known as the bone volume fraction, represents the ratio of the region of interest to the total volume. Tb.sp denotes the average distance of the trabecular structure [63,64]. In our study, BMD and BV/TV were significantly decreased by OVX, as shown in the sagittal, lateral, and cross-sectional images, whereas BT/TV was greater with administration of E_2_, ALN, and TH than that of the OVX group. Tb.sp was greater in the OVX group, and E_2_, ALN, and TH treatment prevented bone loss. Our findings show that E_2_, ALN, and TH treatment inhibited OVX-induced bone loss. Bone is composed of cortical bone and cancellous bone, and the cancellous bone structure is an important factor related to mechanical properties [65]. Histomorphometry is an important method to evaluate the microstructure of bones and can be used to measure the two-dimensional structure of histological sections. H&E staining is the most commonly used staining method to evaluate and measure the trabecular area of the femur [66]. In this study, the trabecular area was lower in the OVX group as compared to the sham group, but in the E_2_, ALN, and TH groups, it was significantly greater. Our findings suggest that TH treatment can prevent OVX-induced decreases in the trabecular area. Immunohistochemical staining is a method used to detect antigens in tissues according to the principle of antibodies binding to antigens in biological tissues [67]. In this study, the expressions of NFATc1 and CTK in bone tissues were confirmed through IHC staining. According to the results of this study, the numbers of NFATc1 and CTK were significantly increased due to OVX, and E_2_, ALN, and TH groups were lower than that of the OVX group. TH significantly inhibited osteoclast differentiation in various experiments, such as TRAP staining and F-actin ring in vitro experiments. In in vivo experiments, TH improved bone density in the OVX-induced model, shown through micro-CT, H&E staining, and IHC staining. Overall, this is a comprehensive study showing that TH has potential as a novel osteoporosis treatment alternative.

However, this study still has some limitations. (I) In the experiment, TH inhibited the expression of NFATc1, c-Fos, and osteoclast-specific gene markers. NF-κB and MAPK are involved in osteoclast differentiation, and the effect of TH on these factors in this study was not elucidated. Further studies are needed to clarify this aspect. (II) We expected that the anti-inflammatory effect of TH would play a positive role in the inhibition of osteoclast differentiation and treatment of osteoporosis, and in fact, TH showed promise as an alternative to the treatment of osteoporosis. However, it is difficult to say that this possibility is based on the anti-inflammatory effect of TH. In the future, verification of the pharmacological effect of TH in animal models of osteoporosis through administration of inflammatory cytokines and in RANKL + TNF-α-induced cell models will be valuable in understanding the anti-osteoporosis effect of TH.

## 5. Conclusions

Overall, this study demonstrated that TH attenuated osteoclast differentiation in vivo and in vitro. TH decreased the expression of the NFATc1/c-Fos pathway, leading to decreased osteoclast differentiation, and TH also improved bone loss in the postmenopausal osteoporosis rat model. TH can lower osteoclast differentiation and prevent osteoporosis; thus, it is a promising candidate herbal medicine for the treatment of postmenopausal osteoporosis.

## Figures and Tables

**Table 1:** Primer sequence for RT-PCR analysis.

Gene Name	Primer Sequence (5′-3′)	Base Pair	Cycle	Temperature	Accession Number
*Mmp9* (MMP-9)	F: CGA CTT TTG TGG TCT TCC CC	258	30	58	NM_013599.4
	R: TGA AGG TTT GGA ATC GAC CC				
*Acp5* (TRAP)	F: ACT TCC CCA GCC CTT ACT ACC G	381	30	58	NM_007388.3
	R: TCA GCA CAT AGC CCA CAC CG				
*Ctks* (CTK)	F: AGG CGG CTA TAT GAC CAC TG	403	26	58	NM_007802.4
	R: CGA CAG CGT CAA ACA AAG GCT TGT A				
*Ca2* (CA2)	F: CTC TCA GGA CAA TGC AGT GCT GA	411	32	58	NM_001357334.1
	R: ATC CAG GTC ACA CAT TCC AGC A				
*Atp6v0d2* (ATP6v0d2)	F: ATG GGG CCT TGC AAA AGA AAT CTG	504	30	58	NM_175406.3
	R: CGA CAG CGT CAA ACA AAG GCT TGT A				
*Oscar* (OSCAR)	F: CTG CTG GTA ACG GAT CAG CTC CCC AGA	310	35	53	NM_001290377.1
	R: CCA AGG AGC CAG AAC CTT CGA AAC T				
*Actb* (β-actin)	F: TTC TAC AAT GAG CTG CGT GT	267	30	58	NM_008084.3
	R: CTC ATA GCT CTT CTC CAG GG				

Abbreviations: MMP-9, matrix metalloproteinase-9; TRAP, tartrate-resistant acid phosphatase; CTK, cathepsin K; CA2, carbonic anhydrase II; ATP6v0d2, ATPase H+ transporting V0 subunit D2; OSCAR, osteoclast-associated immunoglobulin-like receptor.

## Data Availability

Not applicable.
